# Examining CLABSI rates by central-line type

**DOI:** 10.1017/ash.2023.288

**Published:** 2023-09-29

**Authors:** Lauren DiBiase, Shelley Summerlin-Long, Lisa Stancill, Emily Sickbert-Bennett Vavalle, Lisa Teal, David Weber

## Abstract

**Background:** Central-line–associated bloodstream infections (CLABSIs) are linked to increased morbidity and mortality, longer hospital stays, and significantly higher healthcare costs. Infection prevention guidelines recommend line placement in specific insertion locations over others because of the relative risk of infection. The purpose of this study was to assess CLABSI rates by line type to determine whether some central lines had a lower risk of infection and should be recommended over others given similar clinical indications. **Methods:** At UNC Hospitals, data were obtained on central lines across a 3-year period (FY20–FY22) from the EMR (Epic Systems). Central lines were categorized as apheresis catheters, CVC lines (single, double, or triple lumen), hemodialysis catheters, introducer lines, pulmonary artery (PA) catheters, PICC lines (single, double, or triple lumen), port-a-catheters, trialysis catheters, or umbilical lines. The line type(s) associated with each CLABSI during the same period were recorded, and CLABSI rates by line type per 1,000 central-line days were calculated using SAS software. If an infection had >1 central-line device type associated, the infection was counted twice when calculating the CLABSI rate by line type. We calculated 95% CIs for each point estimate to assess for statistically significant differences in rates by line type. **Results:** During FY20–FY22, there were 264,425 central-line days and 458 CLABSIs, for an overall CLABSI rate of 1.73 CLABSIs per 1,000 central-line days. Also, 16% of patients with a CLABSI had >1 type of central line in place. Stratified data on CLABSI rates by each central-line type is presented in the Figure. CLABSI rates were highest in patients with apheresis lines (6.22; 95% CI, 3.96–9.35) and PA catheters (6.22; 95% CI, 3.54–10.20), and the lowest CLABSI rates occurred in patients with PICC lines (1.44; 95% CI, 1.19–1.73) and port-a-catheters (1.14; 95% CI, 0.89, 1.45). For both CVC and PICC lines, as the number of lumens increased from single to triple, CLABSI rates increased, from 0.91 to 2.63 and from 0.57 to 1.20, respectively. **Conclusions:** At our hospital, different types of central lines were associated with statistically higher CLABSI rates. Additionally, a higher number of lumens (triple vs single) in CVC and PICC lines were also associated with statistically higher CLABSI rates. These findings reinforce the importance of considering central-line type and number of lumens to minimize risk of CLABSI while ensuring that patients have the best line type based on their clinical needs.

**Figure:**
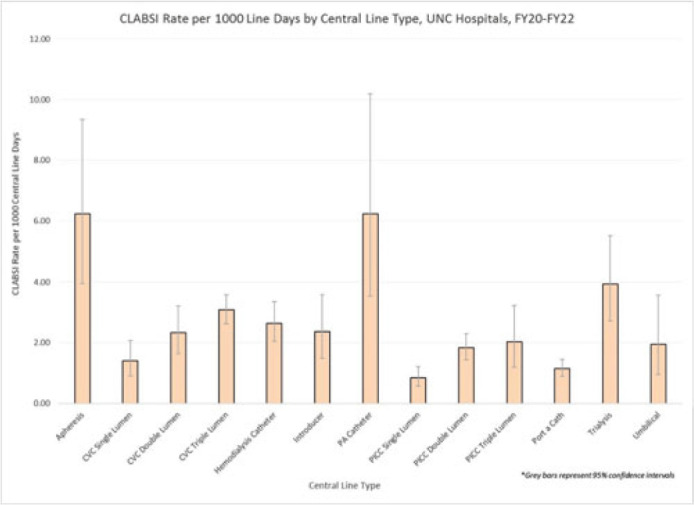

**Disclosures:** None

